# Genome-wide DNA methylation profiling in whole blood reveals epigenetic signatures associated with migraine

**DOI:** 10.1186/s12864-018-4450-2

**Published:** 2018-01-22

**Authors:** Zachary F. Gerring, Allan F. McRae, Grant W. Montgomery, Dale R. Nyholt

**Affiliations:** 1 Statistical and Genomic Epidemiology Laboratory, Institute of Health and Biomedical Innovation, Queensland University of Technology, Brisbane, Queensland Australia; 2 Institute for Molecular Bioscience, The University of Queensland, Brisbane, Queensland Australia; 3 The Centre for Neurogenetics and Statistical Genomics, Queensland Brain Institute, The University of Queensland, Brisbane, Queensland Australia

**Keywords:** Migraine, Differential, Methylation, Blood

## Abstract

**Background:**

Migraine is a common heritable neurovascular disorder typically characterised by episodic attacks of severe pulsating headache and nausea, often accompanied by visual, auditory or other sensory symptoms. Although genome-wide association studies have identified over 40 single nucleotide polymorphisms associated with migraine, there remains uncertainty about the casual genes involved in disease pathogenesis and how their function is regulated.

**Results:**

We performed an epigenome-wide association study, quantifying genome-wide patterns of DNA methylation in 67 migraine cases and 67 controls with a matching age and sex distribution. Association analyses between migraine and methylation probe expression, after adjustment for cell type proportions, indicated an excess of small *P* values, but there was no significant single-probe association after correction for multiple testing (*P* < 1.09 × 10^− 7^). However, utilising a 1 kb sliding window approach to combine adjacent migraine-methylation association P values, we identified 62 independent differentially methylated regions (DMRs) underlying migraine (false discovery rate < 0.05). Migraine association signals were subtle but consistent in effect direction across the length of each DMR. Subsequent analyses showed that the migraine-associated DMRs were enriched in regulatory elements of the genome and were in close proximity to genes involved in solute transportation and haemostasis.

**Conclusions:**

This study represents the first genome-wide analysis of DNA methylation in migraine. We have identified DNA methylation in the whole blood of subjects associated with migraine, highlighting novel loci that provide insight into the biological pathways and mechanisms underlying migraine pathogenesis.

**Electronic supplementary material:**

The online version of this article (10.1186/s12864-018-4450-2) contains supplementary material, which is available to authorized users.

## Background

Migraine is a common disabling, episodic brain disorder, typically characterised by recurring, usually incapacitating attacks of severe pulsating headache and associated symptoms such as nausea, vomiting, photophobia and phonophobia (migraine without aura). In about one-third of patients, attacks are associated with neurological aura symptoms (migraine with aura) [1]. The 2015 Global Burden of Disease (GDB) study estimated the global prevalence of “active migraineurs” (i.e., attacks in the past 12 months) at 13.6% (17.7% of women and 9.5% of men), and is the seventh most disabling disorder in terms of years lost to disability [2]. Migraine has a multifactorial molecular background, driven in part by a highly polygenic mode of inheritance. A recent genome-wide association study (GWAS) of 59,674 migraine cases and 316,078 controls, identified 38 genomic regions harbouring 44 common (minor allele frequency, MAF > 0.01) single nucleotide polymorphism (SNP) loci associated with the disorder [3]. However, the mechanistic role of these loci in migraine pathogenesis is unknown because most reside in non-protein coding regions of the genome that contain tens to hundreds of candidate SNPs in strong linkage disequilibrium (LD) with each other. Multiple lines of evidence suggest GWAS risk variants are enriched in regulatory elements of the genome [4, 5] and modify disease susceptibility by altering the expression levels of their target genes [6–9]. Therefore, the study of non-sequence-based regulatory genomic variation may help elucidate additional factors that influence migraine susceptibility.

Epigenetic processes encompass a variety of chemical and structural modifications to chromosomal regions that mediate the effect of genetic, environmental, and stochastic factors on local transcriptional potential [10]. DNA methylation (5-methylcytosine) is a critical epigenetic process that involves the covalent modification of methyl groups to CpG (5′-cytosine-phosphate-guanine-3′) sites distributed throughout the genome. DNA methylation is dynamic but stably preserved or regenerated from parent to daughter cells [11] and may therefore encode long-lasting changes that mediate expression patterns and disease [12]. The introduction of high-throughput methylation-specific microarray and sequencing technologies has enabled the study of DNA methylation patterns between hundreds to thousands of phenotypically affected cases and controls, known as an Epigenome-wide association study (EWAS). This approach is similar to the study of genetic variability in GWAS, and has identified loci robustly associated, possibly casually, with several complex traits [13–15].

Several lines of evidence support a role for DNA methylation in mediating long-term cellular responses to genetic and environmental effects modifying migraine risk. The heritability for migraine is estimated at around 50% [16–19], indicating both genetic and environmental factors play an important role in its development, and a majority of sufferers report at least one (environmental) trigger factor associated with an attack, including, for example, emotional stress, dietary changes, and a lack of sleep [20]. Attacks of migraine generally begin in early adolescence, are highly episodic with variable symptoms, and peak in frequency during the fourth and fifth decades of life—a period during which work, family, and social environmental stress is highest for many people. Notably, peaks of susceptibility coincide with major hormonal changes in females; many women experience their first migraine around the time of menarche and are more susceptible to attacks during menstruation [21]. Thus, it is reasonable to suggest exposure to environmental risk factors may alter DNA methylation patterns in regulatory elements of genes involved in migraine pathogenesis, thereby lowering the threshold for an attack in predisposed individuals. A genome-wide survey of DNA methylation may identify these epigenetic perturbations and therefore identify novel pathways or help characterise existing risk loci underlying migraine susceptibility.

## Methods

### Brisbane systems genetics study

Participants were recruited from the Brisbane Systems Genetics Study (BSGS), a genetic study of 614 individuals from 314 families of Northern European decent [22]. Families consisted of adolescent monozygotic and dizygotic twin pairs, their non-twin siblings, and their parents. Informed written consent was obtained from each participant, and the study was approved by the Human Research Ethics Committee (HREC) of the QIMR Berghofer (QIMRB) Medical Research Institute.

### DNA methylation pre-processing and normalisation

DNA was extracted from peripheral blood lymphocytes by the salt precipitation method [23]. DNA concentrations were determined by NanoDrop quantification (NanoDrop Techologies, Inc., Wilmington, DE, USA) and standardised to include 500 ng. Three technical replicates were included in each conversion to assess repeatability. Bisulfite conversions were performed in 96 well plates using the EZ-96 DNA Methylation Kit (Zymo Research, Irvine, CA, USA). DNA recovery after conversion was quantified using Nanodrop (Thermo Scientific, Wilmington, DE, USA). Bisulfite converted DNA samples were hybridised to the 12 sample, Illumina HumanMethylation450 BeadChips using the Infinium HD Methylation protocol and Tecan robotics (Illumina, San Diego, CA, USA). Methylation scores for each CpG probe are obtained as a ratio of the intensities of fluorescent signals and are represented as β-values. A threshold for probes showing significant deviation from random missingness was determined by testing against a binomial distribution for the number of samples at the 0.05 significance level with a Bonferroni correction for the number of probes. Any probe with more than 11 individuals with missing data or more than five individuals with detection *P* values > 0.001 were removed. Individual probes were normalised across all samples using a generalised linear model with a logistic link function. Corrections were made for the effects of chip (which encompasses batch processing effects), position on the chip, sex, age, age2, sex × age and sex × age2. After cleaning, 458,835 probes remained. More details regarding pre-processing and normalisation is described in McRae et al. (2014) [24]. DNA methylation data are available at the Gene Expression Omnibus under accession code GSE56105.

### Migraine phenotype

The ID Migraine™ [25] screening questionnaire was used to identify migraine cases. The questionnaire contains three items related to the presence of photophobia, nausea, and health related disability. Respondents with an affirmative response to two or three items were classified as migraine cases. Unrelated controls were selected based on a negative response to ID migraine™ and the absence of family history of migraine. The diagnostic performance of the questionnaire has been validated in a clinical setting [25], and has an estimated sensitivity of 0.84 (95% confidence interval [CI]: 0.75–0.90) and specificity of 0.76 (95% CI: 0.69–0.83). The accuracy of ID migraine™ is similar across sex, age, and the presence of other co-morbid headaches [26], and recent studies have demonstrated clinical utility in adolescents [27, 28].

### Association of DNA methylation values with migraine

Methylation β-values were first adjusted for cell type composition using ReFACTor, a reference-free method for the correction of cell type heterogeneity in DNA methylation studies [29]. The dataset was adjusted for cell composition by regressing out the first five ReFACTor components computed using the default parameters and K = 5. The residualised β-values were then log-transformed to M-values for association testing with migraine. Genome-wide differential methylation analysis between migraine and control groups was performed using generalised linear models with a binomial link function for each of the 458,835 (residualised) methylation probes, adjusted for cohort (i.e., recruitment as an adult or adolescent), using the glm function in R [30]. An ANOVA function was used to test the change in model deviance obtained by adding the methylation value as a covariate. The statistical significance was set at *P* = 0.05 for individual CpGs and *P* = 1 × 10^− 6^ for genome-wide significance, based on a subset of effectively independent methylation probes on the Illumina HumanMethylation450 BeadChip [31].

The global distribution of differentially methylated probes was examined by generating Q-Q plots of *P* values. Q-Q plots were constructed by ranking *P* values in ascending order (the ‘order statistics’) and plotting them against the expected values under the null hypothesis of no association (sampled from the chi-squared distribution). Deviations above the line of equality (shown in white) indicate an excess of smaller *P* values. To aid interpretation we included 95% confidence intervals (shaded in grey), by calculating, for each statistic, the 2.5th and 97.5th centiles of the distribution of the order statistic under random sampling of the null hypothesis. The inflation factor (λ), defined as the ratio of the median observed chi-squared statistic divided by the test statistic of the expected median (0.4549), was also calculated to quantify the excess of smaller *P* values; where the expected λ is 1 under the null hypothesis of no differential methylation.

### Identification and characterisation of differentially methylated regions associated with migraine

Migraine-methylation association *P* values were combined using a 1 kilobase (kb) sliding window, based on the genome-tiling method described by Bock et al. [32]. Methylation probes were first sorted by chromosome and start base pair (bp) position. Based on the start coordinate of each probe and moving consecutively to the last probe on each chromosome, we combined the *P* values from the association analyses for all probes within each window using Fisher’s method. Overlapping regions were merged to form a non-overlapping set of differentially methylated regions. Significant regions were selected at a 5% false discovery rate using the R package qvalue [33]. Differentially methylated regions where methylation values for migraine were less than the controls for the majority of individual CpGs were classified as hypomethylated; while, if the majority of methylation values for migraine were greater less than the controls, they were annotated as hypermethylated.

We also applied the bumphunter technique [34] to identify DMRs. Methylation probes were clustered so that the distance between any two probes in the same cluster was at most 1 kb. For each cluster, a linear model was fit, adjusted for age and sex, and the coefficient for the methylation difference between migraine cases and controls was smoothed using running medians. Each candidate DMR was formally tested for statistical significance by comparing observed methylation differences to a null model generated using 1000 bootstrap samples. The bootstrap-based family-wise error rate (FWER) < 0.1 was used to denote statistical significance [35]. The intersect function of bedtools [36] was used to compare DMRs identified using bumphunter with those identified using our sliding window approach.

### Overlap of differentially methylated regions with genomic features and biological pathways

We examined the overlap between differentially methylated regions and various genomic features, including transcription start sites and gene bodies using Fisher’s exact test, by comparing the proportion of differentially methylated region-associated CpGs overlapping each feature to that of a background list of 458,835 autosomal CpGs from the HumanMethylation450 BeadChip. For biological pathway enrichment analysis, we first mapped differentially methylated regions to their nearest gene, and then tested for the enrichment of genes in Reactome pathways using the g:Profiler web server (http://biit.cs.ut.ee/gprofiler/index.cgi). This program calculates a minimum hypergeometric *P* value for each nominated pathway, placing greater weight on top-ranked genes where enrichment is the strongest, allowing the detection of small and highly significant pathways and larger pathways that are more representative of the entire gene list [37]. We set the minimum overlap between differentially methylated genes and Reactome pathways as *n* = 2, and used a false discovery rate < 0.05 to adjust for multiple testing.

## Results

### Cohort description and migraine phenotype

We identified migraine case and control subjects from the Brisbane Systems Genetics Study (BSGS) using the clinically validated ID migraine™ screening tool [25]. A total of 28 unrelated adults (aged 33–74 years) and 39 unrelated adolescents (aged 12–19 years) screened positive for migraine, and were matched on age and sex with unrelated controls, giving a total sample population of 134 individuals (Table 1).

**Table 1:** Demographic information for individuals with DNA methylation data

	Number (Female)		Mean age (SE)		
Cohort	Cases	Controls	Case	Control	Total
Adults	28 (15)	28 (15)	48.00 (7.90)	46.25 (5.23)	47.12 (6.69)
Adolescents	39 (16)	39 (16)	14.20 (2.36)	14.03 (2.08)	14.12 (2.21)
Total	67 (31)	67 (31)	28.33 (17.63)	27.50 (16.43)	27.91 (16.98)

### Association between migraine status and DNA methylation

**Fig. 1:**
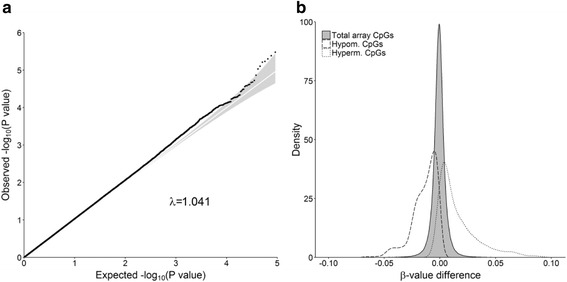
**a** Q-Q plot of –log10 *P* values for differential methylation between migraine cases and non-migraine controls (*N* = 134). **b** Comparison of population level mean β-values for hypomethylated (*n* = 447), hypermethylated (*n* = 244) CpGs, and total array background (*n* = 458,836)

To assess differential methylation between the migraine case and non-migraine control groups, we fitted generalised linear models for each of the 458,835 residualised (i.e., cell type proportion corrected) methylation probes. No single probe achieved genome-wide significance (*P* < 1 × 10^− 6^), however a Q-Q plot showed a greater number of small *P* values than expected under than null hypothesis of no association (i.e., *P* < 0.05 [log_10_(*P*) > 1.3]; λ = 1.041) (Fig. 1a). This suggests many of the differentially methylated probes represent true association signals of small effect, consistent with existing knowledge on regulatory methylation changes underlying complex diseases.

Multiple lines of evidence suggest adjacent methylation values at adjacent CpG sites capture shared regulatory effects [38–40]. As a secondary analysis, we sought to identify extended regions of differential DNA methylation underlying migraine by implementing a recently developed sliding window approach (see Methods). We identified 692 differentially methylated regions which were collapsed to a non-overlapping set of 62 regions, including 45 hypomethylated regions and 17 hypermethylated regions (FDR < 0.05) (Table 2; Additional file 1 Table S1). The average differentially methylated region was 2564 bp in length and contained 11 methylation probes. The methylation value difference between cases and controls for individual probes within each region was small, within the range of 0.005–19.2% (Fig. 1b), but each had concordant effect directions across the length of the region.

**Table 2:** Ten most significant differentially methylated regions associated with migraine

Chromosome	Start (bp)	End (bp)	Direction	*P* value	FDR	No. of Probes	RefSeq gene(s)
11	20,625,538	20,628,781	Decrease	2.54 × 10^− 17^	4.16 × 10^− 12^	10	*SLC6A5*
6	30,033,321	30,046,936	Decrease	1.50 × 10^− 14^	1.23 × 10^− 9^	128	*PPP1R11, RNF39*
9	124,987,896	124,992,432	Increase	1.65 × 10^− 12^	8.99 × 10^−8^	13	*LHX6*
3	185,911,208	185,913,486	Decrease	5.28 × 10^−12^	2.16 × 10^−7^	9	*DGKG*
13	31,506,270	31,508,139	Decrease	2.15 × 10^−11^	7.04 × 10^−7^	11	*TEX26, TEX26-AS1*
11	67,417,958	67,419,405	Decrease	6.54 × 10^−10^	1.79 × 10^− 5^	13	*ACY3*
5	156,886,147	156,888,490	Decrease	1.26 × 10^−9^	2.95 × 10^− 5^	12	*LOC102724404, NIPAL4*
6	74,070,966	74,075,136	Increase	1.99 × 10^−9^	4.08 × 10^− 5^	16	*KHDC3L*
12	52,403,511	52,405,422	Decrease	2.65 × 10^−9^	4.34 × 10^− 5^	6	*GRASP*
13	31,479,366	31,482,184	Decrease	2.46 × 10^−9^	4.34 × 10^−5^	13	*MEDAG, TEX26-AS1*

We identified 9559 candidate DMRs using the bumphunter algorithm, and of these 2683 (28%) had a *P* value < 0.05. A total of 19 candidate regions overlapped the DMRs identified using the sliding window approach, of which 11 (58%) had P value < 0.05 (Additional file 2 Table S2). When the *P* values were compared to a null model such that the family-wise error rate < 0.1, no single region remained significant. The bumphunter region with the smallest FWER adjusted *P* value (FWER = 0.136) was located on chromosome 11, near the gene *ACY3* identified using the sliding window method.

**Fig. 2:**
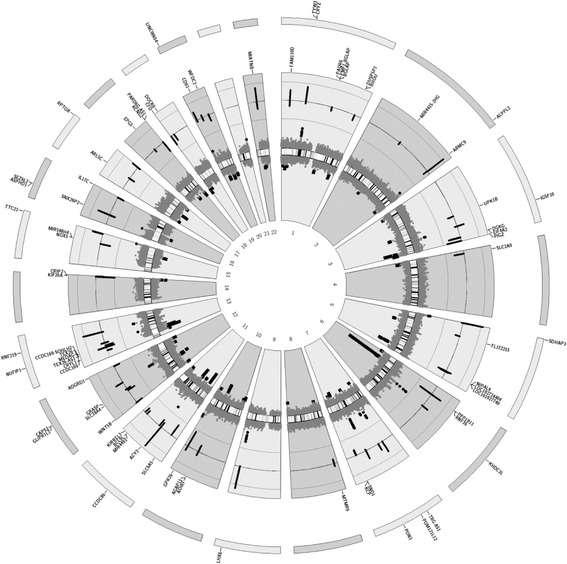
Circos plot of genome-wide DNA methylation changes between migraine cases and non-migraine controls. The inner circle displays an ideogram ordered by chromosome number; black dots represent combined P values using Fisher’s method for each 1-kb sliding region (dots pointing outwards represent hypermethylated windows while the dots pointing inwards represent hypothmetylated regions); green and red dots represent significant (FDR < 0.05) hypermethylated and hypomethylated regions, respecitvely. The middle circle shows the baseline beta-value difference between migraine cases and non-migraine controls for genome-wide signficant regions; the green lines represent hypermethylated regions and red lines hypomethylated regions, and the length of each line represents the beta-value difference. The outermost circles display the RefSeq genes associated with hypomethylated (red) and hypermethylated regions (green)

A genome-wide map of all autosomal CpGs as a circular ideogram, with concentric circles depicting GRCh37/hg19 coordinates and statistical significance denoted by radial orientation and colours, is displayed in Fig. 2. For gene annotation, only those genes within or near genome-wide significant differentially methylated regions (FDR < 0.05) are displayed. The most significant region (FDR = 4.16 × 10^− 12^) was hypomethylated and spanned 10 CpGs within the gene body of *SLC6A5* on chromosome 11 (Table 3). Two other hypomethylated regions overlapped the gene body of solute carrier family-related genes, including *SLC2A9* on chromosome 4 (FDR = 2.59 × 10^− 3^) and *SLC38A4* (FDR = 2.65 × 10^− 3^) on chromosome 12 (Additional file 1 Table S1).

**Table 3:** Association statistics for methylation probes within the most significant differentially methylated region

Summary statistics						Probe expression		Annotation	
Chromosome	Position (bp)	Probe	Coef.	SE	*P* value	β value	Change	Feature	Gene
11	20,625,538	cg07428491	−1.8326	0.8211	0.0208	0.5062	−0.0168	Shelf	*SLC6A5*
11	20,625,714	cg15083015	−1.7232	0.6336	0.0021	0.5019	−0.0318	Shelf	*SLC6A5*
11	20,625,992	cg04968806	−5.1739	1.7130	0.0013	0.5024	−0.0114	Shelf	*SLC6A5*
11	20,626,133	cg21957058	−1.6045	0.6913	0.0149	0.5045	−0.0209	Shelf	*SLC6A5*
11	20,626,264	cg09357935	−1.4053	0.4701	0.0009	0.5031	−0.0453	Shelf	*SLC6A5*
11	20,626,786	cg15828364	−2.1671	0.8315	0.0050	0.5062	−0.0209	Shelf	*SLC6A5*
11	20,627,025	cg23745839	−1.3968	0.5961	0.0146	0.5042	−0.0229	Shelf	*SLC6A5*
11	20,627,327	cg18042724	−2.7853	0.8116	0.0000	0.5069	−0.0374	Shelf	*SLC6A5*
11	20,627,597	cg14524936	−3.6597	1.0867	0.0002	0.5011	−0.0225	Sea	*SLC6A5*
11	20,627,781	cg19172170	−3.0185	1.0297	0.0016	0.5035	−0.0187	Sea	*SLC6A5*

Notes: Coef. denotes the regression coefficient, derived from a general linear model. A negative coefficient indicates lower probe expression in cases relative to controls. β value indicates the mean probe beta value, and change represents the mean difference in probe expression between cases and controls. Feature shows the position of each probe relative to CpG Islands—genomic regions in which the frequency of CpG sites is higher than other regions

### Differentially methylated regions overlap with genomic features and biological pathways

**Fig. 3:**
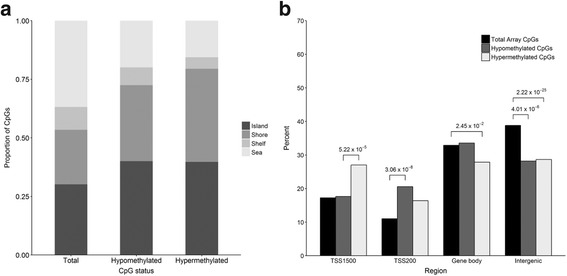
**a** Distribution of CpG sites in CpG islands, shores, shelves, and sea, **b** Distribution of CpG sites relative to transcription start sites, gene bodies, and intergenic regions

We examined the position of differentially methylated regions in relation to CpG islands, RefSeq genes, and various genomic elements involved in the regulation of gene expression. Differentially methylated regions were significantly enriched in CpG islands (*P* = 0.0120) and shores (*P* = 6.79 × 10^− 5^), both sites of transcriptional regulation (Fig. 3a). Hypermethylated regions where enriched in transcription start site sequences located within 1500 bp of RefSeq genes, while hypomethylated regions were enriched in transcription start site sequences located within 200 bp of RefSeq genes. Both hypermethylated and hypomethylated regions were depleted in intergenic regions, with hypermethylated regions also under-represented within gene bodies (Fig. 3b). We also found enrichment of DNase I hypersensitivity sites within hypomethylated and hypermethylated regions (*P* = 2.96 × 10^− 11^). Taken together, these data show migraine-associated regions of methylation are preferentially located within regulatory regions of the genome that may affect the transcriptional activity of nearby genes.

In order to investigate the potential functional consequences of the altered regulatory landscape underlying migraine, we assigned the nearest RefSeq gene(s) to each differentially methylated region and tested for the over-representation of Reactome pathways. While hypermethylated gene lists were not significantly enriched in any biological pathway, genes associated with hypomethylated regions were detected in categories associated with solute transportation (FDR = 0.05; associated genes: *SLC2A9*, *SLC6A5*, and *SLC2A9*) and haemostasis (FDR = 0.084; associated genes: *DGKG*, *KIF26A*, *DOCK6*, *CFD*) (Table 4).

**Table 4:** Pathway analysis of hypomethylated DMR-associated genes in migraine

Reactome pathway	Size	Overlap	FDR	Genes
SLC-mediated transport	272	3	5.00 × 10^−2^	*SLC2A9, SLC38A4, SLC6A5*
Transport of glucose	102	2	5.43 × 10^−2^	*SLC2A9, SLC6A5*
Haemostasis	599	4	8.84 × 10^−2^	*DGKG, KIF26A, DOCK6, CFD*

Notes: Reactome pathway names truncated for presentation purposes. Full pathway annotation ordered by significance: R-HSA-425407 (LC-mediated transmembrane transport); R-HSA-425366 (Transport of glucose and other sugars, bile salts and organic acids, metal ions and amine compounds); R-HSA-109582 (Haemostasis); R-HSA-983231 (Factors involved in megakaryocyte development and platelet production); and R-HSA-382551 (Transmembrane transport of small molecules)

## Discussion

We analysed genome-wide DNA methylation data from migraine cases and non-migraine controls with the aim of identifying differentially methylated regions and pathways underlying the disorder. DNA methylation data measured in whole blood samples collected from 134 unrelated individuals showed global evidence for differential methylation, but no single methylation probe reached genome-wide significance. A sliding window-based approach identified 62 non-overlapping differentially methylated regions significantly enriched in regulatory elements of the genome and in close proximity to genes involved in solute transportation and haemostasis.

Our results should be interpreted within the context of several common limitations associated with case-control studies of DNA methylation. Because methylation patterns can change over an individual’s lifetime in response to genetic, environmental, and stochastic factors, we cannot causally attribute our observed case-control differences to migraine pathogenesis. This is mainly due to the effect of confounding, where an unobserved factor may explain all or part of the association between DNA methylation and migraine. For example, certain medications, such as antidepressants and analgesics, are used more frequently by migraine sufferers than the general population [41] and may also alter methylation patterns. Because the Brisbane Systems Genetics Study did not collect detailed medication use from study participants, we could not adjust for its potential confounding effect. It should be noted, however, that there is little evidence for methylation loci robustly associated with analgesic and antidepressant use in whole blood [42, 43]. Furthermore, to control for confounding and maximise the amount of information per subject studied, we carefully matched cases and controls on age and sex. Nonetheless, unmeasured confounders may still, at least in part, influence our results.

Different tissues, and indeed cell types in the same tissue or organ, tend to exhibit highly characteristic DNA methylation profiles [44]. The choice of tissue for epigenetic studies is therefore an important consideration, as aberrant DNA methylation patterns and their cellular consequences may only be measured in specific tissues. While brain tissue is arguably a critical substrate for epigenetic studies of migraine, it cannot be sampled from living individuals and there is a lack of high quality and well-phenotyped post-mortem samples for molecular dissection. We therefore used whole blood as a surrogate tissue. Blood can be collected from a large number of individuals in a population-based setting [45], and has been used to identify replicable and biologically informative methylation loci underlying susceptibility to several traits [46]. In addition, integrated analyses of SNP genotype and methylation data have found evidence for the co-localisation of existing disease-associated SNP loci and DNA methylation signals in blood and brain [47]. These studies suggest DNA methylation patterns measured in blood may inform disease processes underlying migraine and characterise existing SNP loci identified through GWA studies.

In addition to tissue-specific epigenetic patterns, DNA methylation analyses must also consider the impact of cell-type proportion heterogeneity on study results. Whole blood, for example, contains varying proportions of leukocytes and thrombocytes that contain highly characteristic DNA methylation profiles [48, 49]. Systematic differences in the proportion of these cell types between case and control samples may therefore introduce unwanted variation in DNA methylation and lead to spurious differentially methylated regions. To address this issue, several methods have been developed for estimating cell composition in whole blood [50, 51]. We used a recently developed algorithm based on sparse principal components analysis to remove (unwanted) variation associated with cell type proportions [29].

After adjusting for common confounding variables, including age, sex, and cell subtype proportion heterogeneity, we observed only modest changes in DNA methylation associated with migraine. Furthermore, these changes occurred at intermediate β-value ranges (i.e., β-values between 40% and 60%), rather than the extremes of the scale (˂20% or > 80%). Epigenome-wide association studies of other complex brain-related disorders have reported similarly small changes in methylation that correlated with significant changes in gene expression [13, 15]. The small change in DNA methylation suggests a mosaic subset of cells may contribute to disease susceptibility [52]. The use of single cell techniques to study DNA methylation [53, 54] may therefore be required to experimentally confirm these associations and identify critical cell types involved in disease pathogenesis.

We used a sliding window approach to identify genomic regions associated with migraine, and compared our findings with regions identified using the bumphunter algorithm. Top ranked differentially methylated regions from each method overlapped one another, however the results from bumphunter did not survive correction for multiple testing. While the statistical and conceptual models behind each of these methods are plausible, a systematic benchmarking study is required to draw conclusions on which approach is best for the analysis of complex traits such as migraine. Nonetheless, if there is a power difference between the methods, our results are as expected. That is, our primary sliding window analysis identified significant differentially methylated regions while bumphunter did not, but the top ranked regions from each method overlap. Therefore, we used results from our sliding window approach to further characterise differential methylation underlying migraine.

Differentially methylated regions were enriched in regulatory elements of the genome, including transcription start sites and DNase I hypersensitivity sites, suggesting epigenetic perturbations in migraine are functionally related to transcriptional activity. We classified regions as either hypomethylated or hypermethylated and performed pathway analysis on their nearest gene(s). While genes mapping to hypermethylated regions were not significantly enriched in any particular pathway, most likely due to the low number of mapped genes, hypomethylated regions mapped to genes involved in solute transportation. Three solute transporters were individually associated with hypomethylated regions: *SLC6A5*, a pre-synaptic glycine transporter with an important role in regulating pain perception in inflammatory and neuropathic chronic pain states [55]; *SLC2A9,* a facilitative glucose transporter highly expressed in the kidney cortex with a critical role in regulating blood glucose levels [56]; and *SLC38A4*, a sodium-dependent neutral amino acid transporter highly expressed in hepatic cells [57]. Solute transporters have been linked to several psychiatric disorders that share a higher than expected co-occurrence with migraine, including depression, post-traumatic stress disorder, and anxiety [58]. Aberrant regulation of solute transporters may therefore underlie the co-morbidity between migraine and these disorders. We also found evidence for the enrichment of haemostasis and factors involved in platelet production, an interesting observation given the hypothesised vascular dysfunction in migraine [59], and its co-occurrence with stroke [60] and cardiovascular disease [61].

We relied on the ID migraine™ tool to identify migraine cases and non-migraine controls. The ID migraine™ tool is a clinically validated screening tool with an estimated sensitivity of 81% and specificity of 75%, and has been shown to positively identify 93–98% of people who were subsequently diagnosed with migraine by headache experts using International Headache Society (IHS) criteria [26]. While we believe the ID migraine™ has sufficient accuracy to examine genome-wide DNA methylation in relation to migraine status, future methylomic variation studies should confirm migraine diagnoses using official (i.e. IHS) diagnostic criteria.

Our study lacked power to implicate individual genome-wide significant (*P* < 1.09 × 10^− 6^) CpG sites associated with migraine. For example, power calculations devised by Tsai et al. [31] indicate that at least 100 cases and 100 controls would be required to have 80% power to detect an 11% difference in DNA methylation at genome-wide significance. The recruitment of additional migraine cases and controls would increase power to detect smaller changes in methylation. However, simply increasing sample size within a case-control study design will not mitigate the potential impact of confounding and selection biases on study results. Future studies using alternative study designs may better control these factors. One approach might involve the study of migraine-discordant monozygotic twin pairs, which would remove the influence of common polygenic and many unmeasured environmental factors on the relationship between DNA methylation and migraine. Furthermore, studies of monozygotic twins will improve statistical efficiency by reducing the standard deviation of effect estimates, meaning fewer subjects will be required to detect differential DNA methylation compared to case-control studies. The power and interpretability of future studies may also be improved with the simultaneous measurement of gene expression from the same tissues or cell types, and the integration SNP genotype data from the same individuals. Such an integrated approach may identify the potential cellular consequences of aberrant DNA methylation patterns underlying migraine.

## Conclusions

We identified differentially methylated regions underlying migraine susceptibility. Migraine-associated regions were enriched in regulatory elements of the genome and in close proximity to genes involved in solute transportation and haemostasis. Changes in DNA methylation were subtle but concordant within regions, suggesting the accumulation of many small modifications distributed throughout the genome may influence migraine vulnerability, consistent with a complex model of disease susceptibility. Future studies utilising larger samples and design strategies that appropriately control for unobserved confounding factors, as well as the collection of gene expression and SNP genotype data from the same individuals, will improve the interpretability of DNA methylation patterns underlying migraine and aid the discovery of casual mechanisms and biomarkers.

## Additional files

Additional file 1: Differentially methylated regions identified by a sliding window analysis. This file contains the genomic coordinates for each genome-wide significant differentially methylated region, with additional information on: a) the number of CpGs within each region; b) whether the DMR was hypomethylated or hypermethylated; c) the *P* value and FDR q-value for each DMR; d) the closest gene; e) the beta-value difference for the most significant CpG in the region. (XLSX 19 kb)

Additional file 2: Differentially methylated regions identified using a sliding window approach and the bumphunter algorithm. This additional file contains genomic regions that were differentially methylated between migraine cases and non-migraine controls in both the sliding window and “bumphunter” approach. (XLSX 12 kb)

## References

[1] Launer LJ, Terwindt GM, Ferrari MD (1999). The prevalence and characteristics of migraine in a population-based cohort: the GEM study. Neurology.

[2] Vos T, Barber RM, Bell B, Bertozzi-Villa A, Biryukov S, Bolliger I (2015). Global, regional, and national incidence, prevalence, and years lived with disability for 301 acute and chronic diseases and injuries in 188 countries, 1990-2013: a systematic analysis for the global burden of disease study 2013. Lancet.

[3] Gormley P, Anttila V, Winsvold BS, Palta P, Esko T, Pers TH (2016). Meta-analysis of 375,000 individuals identifies 38 susceptibility loci for migraine. Nat Genet.

[4] Ernst J, Kheradpour P, Mikkelsen TS, Shoresh N, Ward LD, Epstein CB (2011). Mapping and analysis of chromatin state dynamics in nine human cell types. Nature.

[5] Wagner JR, Busche S, Ge B, Kwan T, Pastinen T, Blanchette M (2014). The relationship between DNA methylation, genetic and expression inter-individual variation in untransformed human fibroblasts. Genome Biol.

[6] Emilsson V, Thorleifsson G, Zhang B, Leonardson AS, Zink F, Zhu J (2008). Genetics of gene expression and its effect on disease. Nature.

[7] Nica AC, Montgomery SB, Dimas AS, Stranger BE, Beazley C, Barroso I (2010). Candidate causal regulatory effects by integration of expression QTLs with complex trait genetic associations. PLoS Genet.

[8] Nicolae DL, Gamazon E, Zhang W, Duan S, Dolan ME, Cox NJ (2010). Trait-associated SNPs are more likely to be eQTLs: annotation to enhance discovery from GWAS. PLoS Genet.

[9] Albert FW, Leonid K. The role of regulatory variation in complex traits and disease. Nat Rev Genet. 2015;16. 10.1038/nrg3891 25707927

[10] Bird A (2007). Perceptions of epigenetics. Nature.

[11] Liu X, Gao Q, Li P, Zhao Q, Zhang J, Li J (2013). UHRF1 targets DNMT1 for DNA methylation through cooperative binding of hemi-methylated DNA and methylated H3K9. Nat Commun.

[12] Schubeler D (2015). Function and information content of DNA methylation. Nature.

[13] Huynh JL, Garg P, Thin TH, Yoo S, Dutta R, Trapp BD (2014). Epigenome-wide differences in pathology-free regions of multiple sclerosis-affected brains. Nat Neurosci.

[14] Watson CT, Roussos P, Garg P, Ho DJ, Azam N, Katsel PL (2016). Genome-wide DNA methylation profiling in the superior temporal gyrus reveals epigenetic signatures associated with Alzheimer’s disease. Genome Med.

[15] Jaffe AE, Gao Y, Deep-Soboslay A, Tao R, Hyde TM, Weinberger DR (2016). Mapping DNA methylation across development, genotype and schizophrenia in the human frontal cortex. Nat Neurosci.

[16] Russell MB, Olesen J (1995). Increased familial risk and evidence of genetic factor in migraine. Br Med J.

[17] Ulrich V, Gervil M, Kyvik KO, Olesen J, Russell MB (1999). Evidence of a genetic factor in migraine with aura: a population-based Danish twin study. Ann Neurol.

[18] Gervil M, Ulrich V, Kaprio J, Olesen J, Russell MB (1999). The relative role of genetic and environmental factors in migraine without aura. Neurology.

[19] Mulder EJ, Van Baal C, Gaist D, Kallela M, Kaprio J, Svensson DA (2003). Genetic and environmental influences on migraine: a twin study across six countries. Twin Res.

[20] Kelman L (2007). The triggers or precipitants of the acute migraine attack. Cephalalgia.

[21] Vetvik KG, EA MG, Lundqvist C, Russell MB (2014). Prevalence of menstrual migraine: a population-based study. Cephalalgia.

[22] Powell JE, Henders AK, AF MR, Caracella A, Smith S, Wright MJ (2012). The Brisbane systems genetics study: Genetical genomics meets complex trait genetics. PLoS One.

[23] Miller SA, Dykes DD, Polesky HF (1988). A simple salting out procedure for extracting DNA from human nucleated cells. Nucleic Acids Res.

[24] McRae A, Powell J, Henders A, Bowdler L, Hemani G, Shah S (2014). Contribution of genetic variation to transgenerational inheritance of DNA methylation. Genome Biol.

[25] Lipton RB (2003). A self-administered screener for migraine in primary care: the ID migraine™ validation study. Neurology.

[26] Cousins G, Hijazze S, Van De Laar FA, Fahey T (2011). Diagnostic accuracy of the ID migraine: a systematic review and meta-analysis. Headache.

[27] Zarifoğlu M, Karli N, Taşkapilioğlu Ö (2008). Can ID MigraineTM be used as a screening test for adolescent migraine?. Cephalalgia.

[28] Oztora S, Korkmaz O, Dagdeviren N, Celik Y, Caylan A, Top MS (2011). Migraine headaches among university students using id migraine test as a screening tool. BMC Neurol.

[29] Rahmani E, Zaitlen N, Baran Y, Eng C, Hu D, Galanter J (2016). Sparse PCA corrects for cell type heterogeneity in epigenome-wide association studies. Nat Meth.

[30] R Core Team. R: A Language and Environment for Statistical Computing. 2016. https://www.r-project.org/.

[31] Tsai P-C, Bell JT (2015). Power and sample size estimation for epigenome-wide association scans to detect differential DNA methylation. Int J Epidemiol.

[32] Bock C (2012). Analysing and interpreting DNA methylation data. Nat Rev Genet.

[33] Storey JD, Bass AJ, Dabney A, Robinson D (2015). Qvalue: Q-value estimation for false discovery rate control.

[34] Jaffe AE (2012). Bump hunting to identify differentially methylated regions in epigenetic epidemiology studies. Int J Epidemiol.

[35] Wockner LF, Morris CP, Noble EP, Lawford BR, Whitehall VLJ, Young RM (2015). Brain-specific epigenetic markers of schizophrenia. Transl Psychiatry.

[36] Quinlan AR, Hall IM (2010). BEDTools: a flexible suite of utilities for comparing genomic features. Bioinformatics.

[37] Reimand J, Arak T, Adler P, Kolberg L, Reisberg S, Peterson H (2016). G:profiler—a web server for functional interpretation of gene lists (2016 update). Nucleic Acids Res.

[38] Eckhardt F, Lewin J, Cortese R, Rakyan VK, Attwood J, Burger M (2006). DNA methylation profiling of human chromosomes 6, 20 and 22. Nat Genet.

[39] Bell JT, Pai AA, Pickrell JK, Gaffney DJ, Pique-Regi R, Degner JF, et al. DNA methylation patterns associate with genetic and gene expression variation in HapMap cell lines. Genome Biol. 2011;12 10.1186/gb-2011-12-1-r10 PMC3091299 21251332

[40] Zhang W, Spector TD, Deloukas P, Bell JT, Engelhardt BE (2015). Predicting genome-wide DNA methylation using methylation marks, genomic position, and DNA regulatory elements. Genome Biol.

[41] Stokes M, Becker WJ, Lipton RB, Sullivan SD, Wilcox TK, Wells L (2011). Cost of health care among patients with chronic and episodic migraine in Canada and the USA: results from the international burden of migraine study (IBMS). Headache.

[42] Non AL, Binder AM, Kubzansky LD, Michels KB (2014). Genome-wide DNA methylation in neonates exposed to maternal depression, anxiety, or SSRI medication during pregnancy. Epigenetics.

[43] Wilson LE, Kim S, Xu Z, Harlid S, Sandler DP, Taylor JA (2015). Non-steroidal anti-inflammatory drug use and genomic DNA Methylation in blood. PLoS One.

[44] Dunham I, Kundaje A, Aldred SF, Collins PJ, Davis CA, Doyle F (2012). An integrated encyclopedia of DNA elements in the human genome. Nature.

[45] Gerring Z, Rodriguez-Acevedo AJ, Powell JE, Griffiths LR, Montgomery GW, Nyholt DR (2016). Blood gene expression studies in migraine: potential and caveats. Cephalalgia.

[46] Ligthart S, Marzi C, Aslibekyan S, Mendelson MM, Conneely KN, Tanaka T (2016). DNA methylation signatures of chronic low-grade inflammation are associated with complex diseases. Genome Biol.

[47] Hannon E, Dempster E, Viana J, Burrage J, Smith AR, Macdonald R (2016). An integrated genetic-epigenetic analysis of schizophrenia: evidence for co-localization of genetic associations and differential DNA methylation. Genome Biol.

[48] Dunham I, Kundaje A, Aldred SF, Collins PJ, Davis CA, Doyle F (2012). An integrated encyclopaedia of DNA elements in the human genome. Nature.

[49] Kundaje A, Meuleman W, Ernst J, Bilenky M, Yen A, Roadmap Epigenomics Consortium (2015). Integrative analysis of 111 reference human epigenomes. Nature.

[50] Houseman EA (2012). DNA methylation arrays as surrogate measures of cell mixture distribution. BMC Bioinformatics.

[51] McGregor K, Bernatsky S, Colmegna I, Hudson M, Pastinen T, Labbe A (2016). An evaluation of methods correcting for cell-type heterogeneity in DNA methylation studies. Genome Biol.

[52] Birney E, Smith GD, Greally JM (2016). Epigenome-wide association studies and the interpretation of disease -Omics. PLoS Genet.

[53] Smallwood SA, Lee HJ, Angermueller C, Krueger F, Saadeh H, Peat J (2014). Single-cell genome-wide bisulfite sequencing for assessing epigenetic heterogeneity. Nat Methods.

[54] Farlik M, Sheffield NC, Nuzzo A, Datlinger P, Schönegger A, Klughammer J (2015). Single-cell DNA Methylome sequencing and Bioinformatic inference of Epigenomic cell-state dynamics. Cell Rep.

[55] Foster E, Wildner H, Tudeau L, Haueter S, Ralvenius WT, Jegen M (2015). Targeted ablation, silencing, and activation establish Glycinergic dorsal horn neurons as key components of a spinal gate for pain and itch. Neuron.

[56] Doblado M, Moley KH (2009). Facilitative glucose transporter 9, a unique hexose and urate transporter. Am J Physiol - Endocrinol Metab.

[57] Mackenzie B, Erickson JD (2004). Sodium-coupled neutral amino acid (system N/a) transporters of the SLC38 gene family. Pflügers Arch.

[58] Hahn MK, Blakely RD (2007). The functional impact of SLC6 transporter genetic variation. Annu Rev Pharmacol Toxicol.

[59] Dalkara T, Nozari A, Moskowitz MA (2010). Migraine aura pathophysiology: the role of blood vessels and microembolisation. Lancet Neurol.

[60] Malik R, Freilinger T, Winsvold BS, Anttila V, Vander Heiden J, Traylor M (2015). Shared genetic basis for migraine and ischemic stroke: a genome-wide analysis of common variants. Neurology.

[61] Kurth T, Winter AC, Eliassen AH, Dushkes R, Mukamal KJ, Rimm EB, et al. Migraine and risk of cardiovascular disease in women: prospective cohort study. BMJ. 2016;353 10.1136/bmj.i2610 PMC4887613 27247281

